# Evaluation of Financial Conflicts of Interest and Quality of Evidence Underlying the American Diabetes Association Clinical Practice Guidelines: The Standards of Medical Care in Diabetes, 2021

**DOI:** 10.7759/cureus.36567

**Published:** 2023-03-23

**Authors:** Haruki Shigeta, Anju Murayama, Sae Kamamoto, Hiroaki Saito, Akihiko Ozaki

**Affiliations:** 1 School of Medicine, Tohoku University, Sendai, JPN; 2 School of Medicine, Hamamatsu University School of Medicine, Hamamatsu, JPN; 3 Gastroenterology, Soma Central Hospital, Soma, JPN; 4 Surgery, Teikyo University Graduate School of Public Health, Tokyo, JPN

**Keywords:** diabetes mellitus, clinical practice guideline, physician payment sunshine act, physician payment, open payments database

## Abstract

Background: Clinical practice guidelines make recommendations based on the best available evidence. Proper management and disclosure of financial conflicts of interest (FCOIs) are necessary for trustworthy clinical practice guidelines. This study evaluated the prevalence of FCOIs and quality of evidence underlying the American Diabetes Association (ADA) guidelines.

Methods: Using the Open Payments Database (OPD) between 2018 and 2020, we examined the research and general payments to all authors of the Standards of Medical Care in Diabetes, 2021. The quality of evidence and tone of recommendations were assessed and the associations between the two were evaluated by logistic regression analysis.

Results: Of the 25 guideline authors, 15 (60.0%) were United States (US)-based physicians eligible for the OPD search. Eight (32.0%) and 12 (48.0%) received one or more industry payments one year and three years prior to the guideline publication, respectively. The median total payments (interquartile range) per author were $33,262 ($4,638‒$101,271) in 2020 and $18,053 ($2,529‒$220,659) in 2018-2020. One author received a research payment of over $10,000 undeclared. Of 471 recommendations, 61 (13.0%) and 97 (20.6%) were supported by low-quality evidence and expert opinions, respectively. Also, 439 (93.2%) recommendations had a positive tone. The lower quality of evidence tended to recommend positively with an odds ratio of 1.56 (95% confidence interval: 0.96-2.56, p=0.075) without reaching statistical significance.

Conclusion: A minority of the guideline authors received industry payments from the healthcare industry, and declared FCOIs were mostly accurate. However, the ADA FCOI policy required the guideline authors to declare their FCOIs for one year before publication. A more transparent and rigorous FCOI policy is needed in the ADA guidelines.

## Introduction

Transparency in the management of financial conflicts of interest (FCOIs) and making recommendations based on the best available evidence are the two foundations necessary for developing evidence-based clinical practice guidelines [1]. FCOIs potentially bias recommendations for the benefit of the healthcare industry over patients, as shown in several medical scandals concerning clinical guidelines and clinical trials [2-7]. One systematic review showed that there were associations between authors' conflicts of interest and more favorable recommendations [8]. In the United States (US), diabetes guidelines possibly made more biased recommendations directing pharmacological treatment than guidelines by other associations [9], although the study depended on small sample-sized, self-declared FCOIs.

Additionally, clinical guidelines often made recommendations based on low-quality evidence such as expert opinions and case studies [10-18]. Of the strong recommendations for hepatitis C endorsed by the American Association for the Study of Liver Diseases and Infectious Diseases Society of America, a total of 64.6% were based on a single randomized trial or expert opinion [19]. Of 26 American College of Cardiology/American Heart Association guidelines, only 7.9% of recommendations were supported by high-quality evidence such as multiple randomized clinical trials [10]. Duarte et al. reported that more than half of the recommendations in the American College of Rheumatology practice guidelines were supported by low-quality of evidence [18].

Here, we evaluated FCOIs of the diabetes guidelines issued by the American Diabetes Association (ADA), using the US Open Payments Database, the federal payment disclosure database. Furthermore, we assessed the quality of evidence supporting the recommendations.

## Materials and methods

This study aimed to assess the prevalence of FCOIs and the quality of recommendations of the ADA guideline for diabetes. We considered all guideline authors and recommendations in the Standards of Care in Diabetes, 2021, as this was the latest version of the ADA guidelines for diabetes at the time of the study (November 2021). 

As the ADA guideline disclosed authors’ FCOIs from the past one year and the latest payment data from the Open Payments Database was 2020, we considered all authors of the ADA guideline for diabetes care published in January 2021. Names of authors, quality of evidence, and self-reported conflicts of interest (COIs) were extracted from the guideline. We collected all categories of payments made to ADA guideline authors from the Open Payments Database between January 2018 and December 2020, as many societies require guideline authors to declare their COI for the past three years [12,20]. Payment data of only US-based physicians were available on the Open Payments Database between 2018 and 2020, thus calculation of payments only included US-based physician guideline authors.

Then descriptive analyses were performed on the payment data. Payments per author were calculated based on authors receiving a payment each year, as in other studies [19,21-25]. The ADA guideline classified the quality of evidence into four classes: A (high quality), B (moderate quality), C (low quality), and E (expert opinions). According to the ADA evidence grading system [26], A-quality evidence was defined as clear evidence from randomized controlled trials with sufficient power and generalisability, including evidence from well-conducted multicentre trials and meta-analyses. B-quality evidence included a prospective cohort study or registry and a meta-analysis of cohort studies. C-quality evidence included observational studies with high potential for bias, case series, or case reports. Furthermore, we evaluated the tone of recommendations as positive (encouraging treatment or interventions), negative (discouraging treatment or interventions), or neutral (other) by two independent investigators. Afterward, we calculated the association between the quality of evidence and the tone of recommendation using simple logistic regression analysis.

This study was approved by the Ethics Committee of the Medical Governance Research Institute (approval number: MG2018-04-20200605). Informed consent from the study participants was waived, as this study only analyzed publicly available databases. This study followed the Strengthening the Reporting of Observational Studies in Epidemiology (STROBE) checklist.

## Results

Of the 25 guideline authors, 15 (60.0%) were US-based physicians eligible for the Open Payments Database search (Table 1). Eight (32.0%) and 12 (48.0%) received one or more industry payments one year and three years prior to the guideline publication, respectively. The median total payments (interquartile range (IQR)) per author were $33,262 ($4,638‒$101,271) in 2020 and $18,053 ($2,529‒$220,659) in 2018-2020. In total, eight (32.0%) and 13 (52.0%) authors had financial COIs verified by the Open Payments Database or self-declaration in one year and three years, respectively. One author received a research payment of over $10,000 undeclared. The guideline chairperson received $129 for food and beverage in 2018; however, in the next two years, he did not receive any payments.

**Table 1:** Financial conflicts of interest declared by guideline authors and verified by the Open Payments Database *Payments per author were calculated among the guideline authors with industry payments, as majority of authors did not receive payments. **Number of guideline authors with FCOIs verified by authors’ self-declaration in the guideline or the Open Payments Database between January 2018 and December 2020. COI: conflict of interest; NA: not available; IQR: interquartile range; FCOI: financial conflict of interest

Variables	Guideline authors (n=25)		Acknowledged authors (n=13)		Overall (n= 38)	
	In 2020	2018‒2020	In 2020	2018‒2020	In 2020	2018‒2020
Authors’ self-declared COI with industry, n (%)	9 (36.0)	NA	NA		NA	NA
Open Payments Database-based COI						
Number of eligible authors for Open Payments search, n (%)	15 (60.0)		9 (69.2)		24 (63.2)	
Number of authors with industry payments, n (%)						
General payments	8 (32.0)	12 (48.0)	3 (23.1)	8 (61.5)	11 (28.9)	20 (52.6)
Associate research payments	2 (8.0)	4 (16.0)	3 (23.1)	3 (23.1)	5 (13.2)	7 (18.4)
Research payments	4 (16.0)	12 (48.0)	4 (30.8)	2 (15.4)	8 (21.1)	6 (15.8)
All categories	8 (32.0)	12 (48.0)	6 (46.2)	8 (61.5)	14 (36.8)	20 (52.6)
Total amounts by category, USD						
General payments	411,572	1,812,991	77,544	474,008	489,116	2,286,999
Associate research payments	14,087,019	21,035,426	148,718	228,227	14,235,737	21,263,653
Research payments	30,230	77,092	30,418	26,795	60,648	103,887
All categories	14,528,821	22,925,510	256,681	729,029	14,785,501	23,654,540
Median payments per author (IQR), USD*						
General payments	31,062 (4,638‒67,441)	12,982 (2,529‒194,222)	16,226 (9,976‒36,910)	30,328 (8,129‒222,929 )	22,890 (4,442‒61,461)	12,117 (2,731‒135,212)
Associate research payments	7,043,509 (3,578,526‒10,508,493)	205,474 (35,144‒5,429,187)	11,271 (6,131‒73,863)	140,760 (46,201‒364,747 )	113,543 (11,271‒136,455)	79,860 (26,903‒252,754)
Research payments	6,489 (4,400‒9,646 )	15,651 (9,975‒$24,949)	5,740 (2,585‒10,760)	11,400 (5,700‒19,901)	6,489 (4,025‒9,649)	15,651 (7,125‒23,584)
All categories	33,262 (4,638‒101,271)	18,053 (2,529‒220,659)	6,153 (3,106‒66,767)	11,988 (5,547‒74,814)	17,935 (3,806‒82,054)	12,853 (2,731‒169,205)
Number of authors with COI**	9 (36.0)	13 (52.0)	9 (69.2)		18 (47.4)	22 (57.9)

Among 471 overall recommendations, 61 (13.0%) and 97 (20.6%) were supported by low-quality evidence and expert opinions, respectively, while 29.3% were based on high-quality evidence including well-controlled multicenter clinical trials and meta-analyses. Also, 439 (93.2%) recommendations had positive tones (Table 2). The lower quality of evidence tended to recommend positively with an odds ratio of 1.56 (95%CI: 0.96-2.56, p=0.075) but did not reach statistical significance. Limiting the recommendation to pharmacotherapy revealed the lower quality of evidence tends to recommend positively with an odds ratio of 1.61 (95%CI: 0.75 - 3.49, p=0.222), which also did not reach statistical significance.

**Table 2:** Quality of evidence and tone of recommendations * The ADA guidelines provided four categories of quality of evidence on which guideline recommendations are based, and the levels described in the guidelines were used in this analysis. ** The tone of recommendations was classified into three categories: positive (recommendations encouraging treatment, interventions, and testing), negative (discouraging treatment, interventions, and testing), and neutral (other recommendations) by two independent investigators. ADA: American Diabetes Association

Quality of evidence, n (%)*	Tone of recommendation**		
	Positive	Neutral	Negative
A (Large well-designed clinical trials or well-done meta-analyses)	126 (26.8)	2 (0.4)	10 (2.1)
B (Well-conducted cohort or case-controlled studies)	164 (34.8)	4 (0.8)	7 (1.5)
C (Poorly controlled or uncontrolled observational studies and care series)	56 (11.9)	2 (0.4)	3 (0.6)
E (Expert consensus or clinical experience)	94 (20.0)	1 (0.2)	2 (0.4)

## Discussion

This study elucidated eight (32.0%) ADA guideline authors had FCOIs with industry and FCOIs were majorly consistent with payment data in the one year before the guideline publication. Only one author failed to self-report FCOI. Many previous studies showed that there were widespread financial conflicts of interest between guideline authors and the healthcare industry in most specialties [13,16,17,19,27-37]. Most of these studies reported that the majority of the guideline authors received payments from the healthcare industry [3,38-43] and there were large amounts of undisclosed FCOIs with the healthcare industry [13,16,17,34,38,44]. Compared to these studies, our findings indicate that the authors’ self-declared FCOIs were mostly accurate and the high integrity of the ADA guideline authors.

However, in the past three years, more than 50% of the guideline authors had FCOIs with industry, which was a deviation from the US Academy of Medicine’s trustworthy guideline standards [1]. The Internal Committee of Medical Journal Editors (ICMJE) and many clinical practice guideline-developing organizations including the American Gastroenterological Association (AGA) and the American Society of Clinical Oncology (ASCO) have expanded the FCOI declaration policy to a longer lookback period such as three years before publication [12,20]. As our study showed, the shorter lookback period underestimated the magnitude of FCOIs among guideline authors, though the shorter ADA COI declaration period might have led to a more accurate COI declaration than other clinical guidelines previously documented [13,17,29,31,34,38,44-46]. However, longer look-back periods, such as three years prior to guideline publication, were established based on the many previous cases of potential harm to patients caused by FCOIs and the increased public demand for greater transparency [1,4,5]. The current ADA policy of self-declarations largely relied on author integrity and this system underestimates the magnitude of FCOIs as one author received undeclared research payment from a pharmaceutical company. Cross-checking and verification by a public payment database such as the Open Payments Database would increase the accuracy of FCOI disclosures and assist guideline authors in their FCOI declaration.

Besides FCOI, greater than one-third of clinical practice guideline recommendations were based on low-quality evidence with almost all positive tones. Importantly, recommendations supported by lower-quality evidence might encourage more intervention. When high-quality evidence was not available, experts’ opinions and consensus-based recommendations were made. However, expert-consensus-based recommendations gravitate towards discordant recommendations than evidence-based approaches [47]. Additionally, the clinical guideline should make balanced recommendations, and should not be a short list of dos and don’ts [1]. Therefore, well-balanced, higher-quality, evidence-based diabetes guidelines are essential, in addition to a more rigorous FCOI management. Increased transparency is needed in the Open Payments Database with database-covered recipients expanded to nurse practitioners and physician assistants from 2021 payment data. We believe the timing is appropriate to reform the ADA COI policy for the declaration period and utilization of the Open Payments Database.

This study included several limitations. First, we could not exclude the possibility of errors in the Open Payments Database, as some previous studies [48] with much larger sample sizes found there were errors in payment data. However, physicians can review and dispute their payment records prior to data release (as the Open Payment Database may contain errors in payment data) but very few physicians disputed. We took this to indicate that the Open Payment Database is highly accurate and reliable. Second, at the time of this study, the Open Payments Database only covered payments to physicians from the pharmaceutical and medical device companies manufacturing medical products approved by the US Food and Drug Administration. Therefore, there would be unmeasured financial relationships between the ADA guideline authors and the healthcare industry not covered by the Open Payments. Third, our findings may not be generalized to ADA guidelines published in other years.

## Conclusions

A minority of the guideline authors received industry payments from the healthcare industry one year before the guideline publication. These authors’ declared FCOIs were mostly accurate. However, the ADA FCOI policy set a shorter period for authors’ FCOI declaration than other global FCOI policies. More than 50% of the guideline authors had FCOIs three years before the publication. A more transparent and rigorous FCOI policy is needed in the ADA guidelines.
